# FGF1^ΔHBS^ prevents diabetic cardiomyopathy by maintaining mitochondrial homeostasis and reducing oxidative stress via AMPK/Nur77 suppression

**DOI:** 10.1038/s41392-021-00542-2

**Published:** 2021-03-24

**Authors:** Dezhong Wang, Yuan Yin, Shuyi Wang, Tianyang Zhao, Fanghua Gong, Yushuo Zhao, Beibei Wang, Yuli Huang, Zizhao Cheng, Guanghui Zhu, Zengshou Wang, Yang Wang, Jun Ren, Guang Liang, Xiaokun Li, Zhifeng Huang

**Affiliations:** 1 School of Life and Environmental Science, Wenzhou University, Wenzhou, Zhejiang China; 2 School of Pharmaceutical Sciences and Center for Structural Biology, Wenzhou Medical University, Wenzhou, Zhejiang China; 3 The 2nd Affiliated Hospital, Wenzhou Medical University, Wenzhou, Zhejiang China; 4 Department of Cardiology and Shanghai Institute of Cardiovascular Diseases, Zhongshan Hospital Fudan University, Shanghai, China

**Keywords:** Drug discovery, Endocrine system and metabolic diseases, Cardiology

## Abstract

As a classically known mitogen, fibroblast growth factor 1 (FGF1) has been found to exert other pleiotropic functions such as metabolic regulation and myocardial protection. Here, we show that serum levels of FGF1 were decreased and positively correlated with fraction shortening in diabetic cardiomyopathy (DCM) patients, indicating that FGF1 is a potential therapeutic target for DCM. We found that treatment with a FGF1 variant (FGF1^∆HBS^) with reduced proliferative potency prevented diabetes-induced cardiac injury and remodeling and restored cardiac function. RNA-Seq results obtained from the cardiac tissues of *db/db* mice showed significant increase in the expression levels of anti-oxidative genes and decrease of *Nur77* by FGF1^∆HBS^ treatment. Both in vivo and in vitro studies indicate that FGF1^∆HBS^ exerted these beneficial effects by markedly reducing mitochondrial fragmentation, reactive oxygen species (ROS) generation and cytochrome c leakage and enhancing mitochondrial respiration rate and β-oxidation in a 5’ AMP-activated protein kinase (AMPK)/Nur77-dependent manner, all of which were not observed in the AMPK null mice. The favorable metabolic activity and reduced proliferative properties of FGF1^∆HBS^ testify to its promising potential for use in the treatment of DCM and other metabolic disorders.

## Introduction

Diabetic cardiomyopathy (DCM) is a series of changes in the myocardial structure and function caused by diabetes mellitus (DM) that are not related to coronary atherosclerosis, hypertension, and valvular heart disease.^1,2^ Currently, DCM is the main cause of heart failure and death in DM patients due to no effective therapy. In the setting of diabetes, a metabolic shift in cardiomyocytes occurred due to insulin resistance or lack of insulin, whereby fatty acid uptake and β-oxidation are increased to maintain ATP production. However, with disease progressing, the incoming fatty acids cannot be adequately metabolized through β-oxidation, resulting in intracellular lipid accumulation and lipotoxicity, which induces mitochondrial dysfunction and overproduction of reactive oxygen species (ROS) in cardiomyocytes.^1,3^ Finally, these effects cause cardiomyocyte death, cardiac hypertrophy, inflammation and fibrotic remodeling.^1^ Therefore, maintaining cardiac metabolic homeostasis is a promising therapeutic strategy for DCM.

Fibroblast growth factor 1 (FGF1), the founding member of the FGF family, is well known for its mitogenic activity on cells from a variety of tissue origins, including liver, vasculature, and skin.^4–6^ Recently, FGF1 was identified as an unexpected metabolic hormone playing a pivotal role in the regulation of insulin sensitivity, glycemic control, and nutrient stress.^7–9^ Interestingly, it also displayed therapeutic effects on diabetic nephropathy^10^ and favorable effects on maintaining myocardial integrity and preventing cardiac dysfunction in the setting of diabetes or post-myocardial infarction (MI).^11–13^ Therefore, FGF1 has great potency in the prevention and treatment of DCM. However, long-term use of wild-type FGF1 (FGF1^WT^) may increase tumorigenic risks because of its strong mitogenic activity, which limits the applications of FGF1^WT^ in vivo, especially in cancer-prone diseases including diabetes.^7^ It has been reported that FGF1 is involved in maintaining the metabolic homeostasis of adipose tissue under variations of nutrient availability.^14^ However, the roles of FGF1 or FGF1 variants in cardiac metabolic homeostasis have not been studied.

To address these issues, we have recently generated a novel FGF1 variant that diminished its ability to induce heparan sulfate (HS)-assisted FGF receptor (FGFR) dimerization and activation by replacing three key residues from the HS-binding site of FGF1 with residues that are less optimal for HS-binding (i.e., Lys127Asp, Lys128Gln and Lys133Val; termed FGF1^ΔHBS^), which exhibits full metabolic capacity but much lower proliferative potency compared to FGF1^WT^.^15^ To test the protective role and underlying mechanism of FGF1^ΔHBS^ against DCM, murine models of type 2 diabetes were employed and we found that FGF1^∆HBS^ treatment prevented DCM with inhibition of cardiac hypertrophy, fibrosis, dysfunction, and preserved metabolic homeostasis. The underlying mechanisms involved cardiac 5’ AMP-activated protein kinase (AMPK)-mediated inhibition of Nur77 expression and mitochondrial translocation that prevented diabetes-induced cardiomyocyte mitochondrial fragmentation and oxidative stress.

## Results

### Diminished FGF1 expression in DCM patients and *db/db* mice

**Fig. 1:**
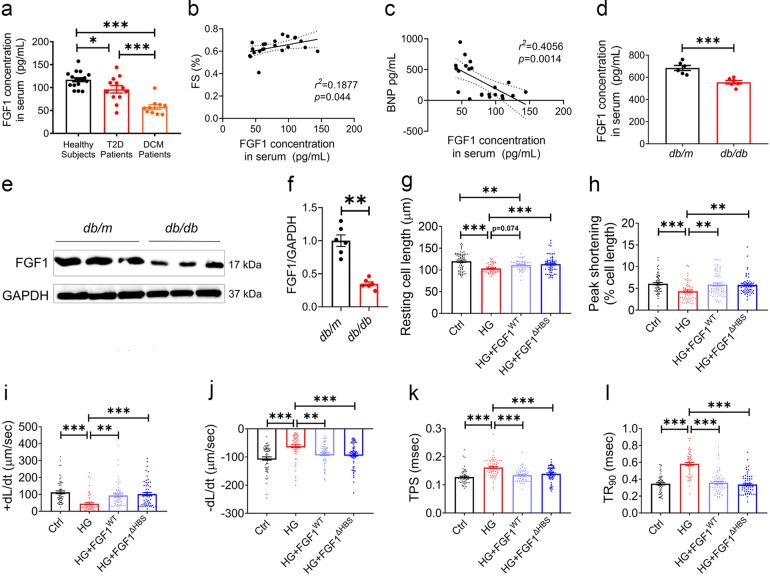
Decreased endogenous FGF1 levels in diabetic individuals and comparably protective function of FGF1^WT^ and FGF1^ΔHBS^ in high-glucose-treated cardiomyocytes. **a** Serum levels of FGF1 in healthy subjects (*n* = 17), T2D patients with (*n* = 10) and without (*n* = 12) DCM. **b** Correlation between serum FGF1 levels and fractional shortening (FS) in T2D patients. **c** Correlation between serum FGF1 and BNP levels in T2D patients. **d** Serum FGF1 levels in *db/m* (Ctrl) and *db/db* (T2D) mice determined by ELISA. *n* = 6. **e** Representative western blot analysis of FGF1 in cardiac tissues from *db/m* and *db/db* mice. GAPDH was a loading control. **f** Densitometric quantification of western blots as shown in **e**. *n* = 6. **g**–**l** Contractile properties of primary cardiomyocytes from adult C57Bl/6J mice were treated with FGF1^WT^ or FGF1^ΔHBS^ (500 ng/mL for 1 h) and exposed to high glucose (HG, 35 mM) for 5 h. *n* = 62–65. **g** Resting cell length. **h** Peak shortening normalized to resting cell length. **i** Maximal velocity of shortening (+d*L*/d*t*). **j** Maximal velocity of re-lengthening (−d*L*/d*t*). **k** Time to peak shortening. **l** Time to 90% re-lengthening. Data were mean ± SEM; *P < 0.05, **P < 0.01, ***P < 0.001

Compared to healthy controls, serum FGF1 levels were decreased in T2D and DCM patients. There was a ~40% reduction in serum FGF1 levels in the patients with DCM compared to the patients without DCM (Fig. 1a), and serum FGF1 levels were positively correlated with the fractional shortening (FS) (Fig. 1b) and negatively correlated with the serum brain natriuretic peptide (BNP) (Fig. 1c). Consistent with patient data, serum and cardiac tissue levels of FGF1 in T2D mice (*db/db*) were decreased by ~20% (Fig. 1d) and ~60% (Fig. 1e, f) relative to the control group (*db/m*), respectively.

### Non-mitogenic FGF1^ΔHBS^ preserved the protective effect on cardiomyocytes of FGF1^WT^

The protective effects of the FGF1^△HBS^ and FGF1^WT^ were compared in high glucose (HG) challenged primary adult mouse cardiomyocytes. HG reduced resting cell length, peak shortening (PS) and maximal velocity of shortening and re-lengthening (±d*L*/d*t*) accompanied with prolonged time-to-PS (TPS) and time-to-90% re-lengthening (TR_90_) (Fig. 1g–l). FGF1^WT^ and FGF1^ΔHBS^ comparably protected the cardiomyocytes against HG-induced mechanical dysfunction (Fig. 1g–l). Furthermore, HG-induced cardiomyocyte hypertrophy and apoptosis were comparably prevented by FGF1^WT^ and FGF1^ΔHBS^ treatment (Supplemental Fig. S1). These data suggest comparably beneficial roles of FGF1^ΔHBS^ and FGF1^WT^ in HG-treated cardiomyocytes.

### Long-term treatment of FGF1^∆HBS^ prevented DCM in *db/db* mice

**Fig. 2:**
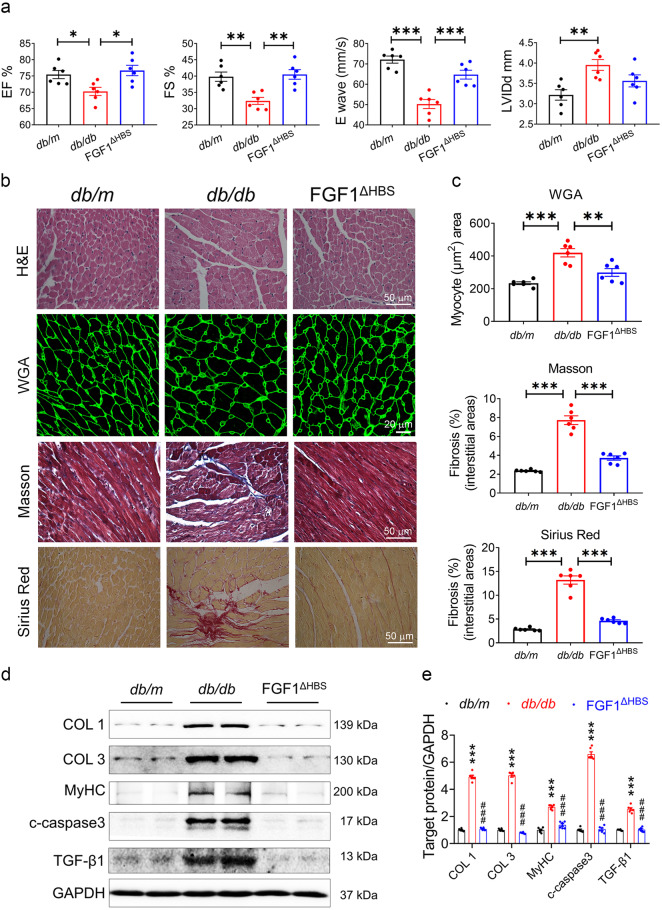
FGF1^ΔHBS^ prevents cardiac dysfunction and remodeling in *db/db* mice. **a**–**e** *db/db* mice were treated with FGF1^ΔHBS^ (0.5 mg/kg body weight) or vehicle every other day for 16 weeks, and littermate *db/m* mice served as controls. **a** Echocardiographic parameters. *n* = 6. **b** Representative images of hematoxylin-eosin (H&E), WGA, Masson’s trichrome, Sirius red staining in cardiac tissues. **c** Quantification of myocyte area and cardiac fibrosis area in WGA, Masson’s trichrome and Sirius Red staining. *n* = 6. **d** Western blot analysis of collagen I (COL 1), collagen III (COL 3), myosin heavy chain (MyHC), cleaved caspase 3 (c-caspase 3) and transforming growth factor β1 (TGF-β1) in cardiac tissues; GAPDH was a loading control. **e** Densitometric quantification of western blots shown in **d**. *n* = 6. Data were mean ± SEM; *P < 0.05, **P < 0.01, ***P < 0.001 in **a**, **c**; ***P < 0.001 vs. *db/m*, ^###^*P* < 0.001 vs. *db/db* in **e**

We then explored the protective effect of FGF1^∆HBS^ in *db/db* mice by intraperitoneal injection of recombinant human FGF1^∆HBS^ (0.5 mg/kg body weight) on alternate days over 16 weeks. Cardiac systolic and diastolic dysfunctions were observed in the *db/db* mice evidenced by reduced EF%, FS%, and E Wave, as well as increased LVIDd, IVSd and PWd, and these unfavorable changes of the heart were mitigated with FGF1^∆HBS^ treatment (Fig. 2a and Supplemental Table S1). The impaired IRT and Tei index of the *db/db* mice were improved with FGF1^∆HBS^ treatment as well (Supplemental Table S1). Meanwhile, FGF1^∆HBS^ treatment also decreased the levels of serum CK-MB, blood glucose and insulin of the *db/db* mice (Supplemental Table S1 and Supplemental Fig. S2a). Furthermore, the blood glucose levels of *db/db* mice treated with FGF1^△HBS^ remained lower throughout the glucose tolerance test (GTT) (Supplemental Fig. S2b).

DCM is characterized by structural and functional derangement of cardiac muscles, including myocardial fibrosis, hypertrophy and contractile dysfunction.^2^ Hence, H&E and wheat germ agglutinin (WGA) staining showed myofiber disarray and cardiomyocyte hypertrophy in *db/db* mice, which were improved with FGF1^ΔHBS^ treatment (Fig. 2b, c). Further Masson trichrome and Sirius red staining showed significant cardiac fibrosis in the *db/db* mice that was prevented with FGF1^ΔHBS^ treatment (Fig. 2b, c and Supplemental Fig. S2c). Western blot analysis showed 2- to 4-fold increases in collagen I (COL 1), collagen III (COL 3) and myosin heavy chain (MyHC) proteins in *db/db* mice, and these were completely reversed with FGF1^ΔHBS^ treatment (Fig. 2d, e). Similarly, FGF1^ΔHBS^ treatment significantly ameliorated the increased transforming growth factor β1 (TGF-β1) and cleaved caspase 3 (c-caspase 3) levels in the *db/db* mice (Fig. 2d, e). These data suggest that FGF1^∆HBS^ mitigates myocardial remodeling, systolic and diastolic dysfunction in diabetic mice.

### FGF1^∆HBS^ reduced oxidative stress in the cardiac tissues of *db/db* mice

**Fig. 3:**
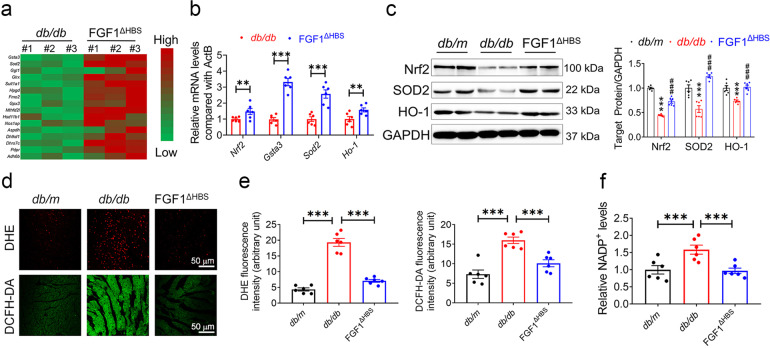
FGF1^ΔHBS^ preserves anti-oxidant capacity in the hearts of *db/db* mice. **a** Hierarchical clustering of FGF1^ΔHBS^-upregulated genes related to anti-oxidative stress based on the RNA sequencing analysis in the hearts from from *db/db* and *db/db* + FGF1^ΔHBS^ mice. *n* = 3. **b** The mRNA levels of Nrf2 and its target genes *Gsta3*, *Sod-2* and *Ho-1* in the cardiac tissues. *n* = 6. **c** Western blot analysis (left panel) and densitometric quantification (right panel) of Nrf2, SOD2 and HO-1 in the cardiac tissues. GAPDH was a loading control. *n* = 6. **d** Representative images of DHE and DCFH-DA staining in the cardiac tissues. **e** Quantification of the corresponding fluorescence intensity in **d**. *n* = 6. **f** Relative concentration of NADP^+^ normalized to the *db/m* group. *n* = 6. Data were mean ± SEM; **P < 0.01 ***P < 0.001 in (**b**, **e**, **f**); ***P < 0.001 vs. *db/m*, ^###^*P* < 0.001 vs *db/db* in **c**

To explore the underlying mechanism of FGF1^ΔHBS^ in preventing DCM, RNA sequencing (RNA-Seq) analysis was conducted. Compared to the vehicle treatment group, multiple genes associated with anti-oxidative signaling (i.e., glutathione S-transferase α3 (*Gsta3*), superoxide dismutase-2 (*Sod2*) and heme oxygenase 1 (*Ho-1*)) were significantly upregulated by FGF1^ΔHBS^ treatment (Fig. 3a). The differential induction of gene expression observed via RNA-Seq was verified by real-time PCR analyses (Fig. 3b). Further western blot showed that protein levels of nuclear factor erythroid-2-related factor 2 (Nrf2), SOD2 and HO-1 were suppressed in the cardiac tissues from *db/db* mice, which were significantly restored with FGF1^ΔHBS^ treatment (Fig. 3c). ROS level was examined using DHE and DCFH-DA probes in cardiac tissues. The results showed higher fluorescence intensities of DHE and DCFH-DA in the cardiac tissues of *db/db* mice compared to *db/m* mice, which were reduced by FGF1^ΔHBS^ treatment (Fig. 3d, e). Similarly, increased NADP^+^ content in the cardiac tissues of *db/db* mice was ameliorated with FGF1^ΔHBS^ treatment (Fig. 3f). These data suggest that FGF1^ΔHBS^ treatment prevents cardiac cells from oxidative stress in *db/db* mice.

### FGF1^∆HBS^ prevented mitochondrial fission and dysfunction in *db/db* mouse heart

**Fig. 4:**
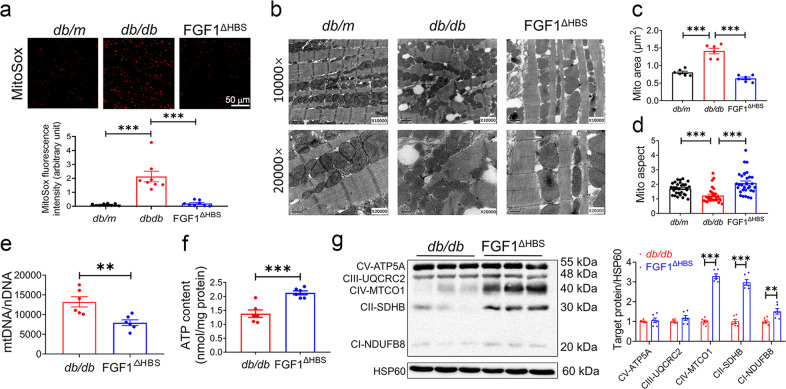
FGF1^ΔHBS^ prevents mitochondrial ROS production and dysfunction in the hearts of *db/db* mice. **a** Representative images (upper panel) and quantification (lower panel) of fluorescence intensity of MitoSox in cardiac tissues from *db/db* and *db/db* + FGF1^ΔHBS^ mice. *n* = 6. **b** Representative transmission electron micrographs of cardiac tissues in each group. **c** Mitochondrial area in cardiac tissues of each group. *n* = 6. **d** Mitochondrial aspect ratio (long/short axis) in the cardiac tissues. *n* = 30–31. **e** mtDNA copy number per nuclear genome in the cardiac tissues. *n* = 6. **f** ATP content in the cardiac tissues. *n* = 6. **g** Western blot analysis (left panel) and densitometric quantification (right panel) of mitochondrial respiratory complex in the cardiac tissues. HSP60 was a loading control. *n* = 6. Data were mean ± SEM; **P < 0.01, ***P < 0.001

Mitochondria-derived anions are the major intracellular source of ROS in diabetes.^16^ Our data showed increased mitochondrial ROS (mROS) in the cardiac tissues from *db/db* mice, which was reduced by FGF1^ΔHBS^ treatment as manifested by fewer MitoSox-positive cells (Fig. 4a). Several lines of evidence noted accumulation of fragmented mitochondria in hyperglycemic patients and animals, indicating a role for abnormal fission in mitochondrial dysfunction and ROS production.^17,18^ Ultrastructural examination in cardiac tissues revealed massive and irregular accumulation of mitochondria with irregular myofilament array in the hearts of *db/db* mice, and FGF1^ΔHBS^-treated *db/db* mice displayed fewer number and enlarged size of mitochondria with a restored aspect ratio to the level of the control mice (Fig. 4b–d). Remarkably, cardiomyocytes from *db/db* mice contains ~13,000 copies of mtDNA per nuclear genome, whereas there were 7800 copies of mtDNA per nuclear genome in the cardiomyocytes from FGF1^ΔHBS^-treated mice (Fig. 4e). As shown in Fig. 4f, cardiac tissues from FGF1^ΔHBS^-treated *db/db* mice had higher ATP content compared to those of the vehicle-treated mice. In consistent, proteins of respiratory complexes, including complex I, II, and IV were upregulated with FGF1^ΔHBS^ treatment (Fig. 4g).

The results from RNA-Seq and real-time PCR also confirmed that FGF1^ΔHBS^ treatment ameliorated mitochondrial dysfunction in *db/db* mice (Supplemental Fig. S3). First, genes involved in electron transport chain and mitochondria biogenesis were analyzed based on RNA-Seq results (Supplemental Fig. S3a, b). FGF1^ΔHBS^ treatment significantly increased the levels of *Uqcrc1, Cox4i1*, *Nrf1 and Pgc1α* (Supplemental Fig. S3b). In consistence with increased ATP production and gene expression of OXPHOS (oxidative phosphorylation) units, the mRNA levels of electron transfer units and mediators of uncoupling such as *Etfa*, *Etfb*, *Etfdh*, *Ucp2*, *Ucp3* and *Slc25a4* were upregulated by FGF1^ΔHBS^ (Supplemental Fig. S3c, d). Taken together, ultrastructural, immunofluorescent and functional data supported a beneficial role of FGF1^ΔHBS^ in suppressing mitochondrial fission and dysfunction in diabetic hearts.

### AMPK-mediated Nur77 suppression contributed to the protective role of FGF1^∆HBS^ against DCM

**Fig. 5:**
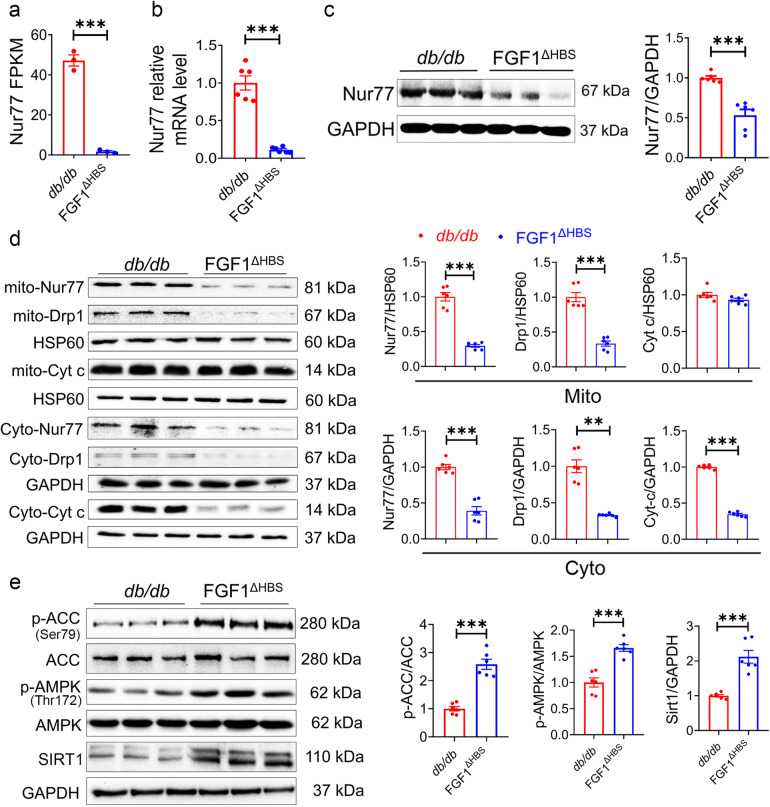
FGF1^ΔHBS^ suppresses Nur77 expression and activates AMPK in the hearts of *db/db* mice. **a** FPKM values of Nur77 gene in the RNA-Seq analysis. *n* = 3. **b** Nur77 mRNA levels confirmed by qPCR. *n* = 6. **c** Western blot analysis (left panel) and densitometric quantification (right panel) of Nur77 in the cardiac tissues. GAPDH was a loading control. *n* = 6. **d** Western blot analysis (left panel) and densitometric quantification (right panel) of Drp1, Nur77 and Cyt C in cardiac tissues of each group. HSP60 and GAPDH were loading controls. *n* = 6. **e** Western blot analysis (left panel) and densitometric quantification (right panel) of p-ACC, ACC, p-AMPK, AMPKα2 and SIRT1 in the cardiac tissues. GAPDH was a loading control. *n* = 6. Data were mean ± SEM; **P < 0.01, ***P < 0.001

The RNA-Seq results showed that mRNA levels of *Nur77* was downregulated by ~20-fold (Fig. 5a), which plays a critical role in causing mitochondrial dysfunction in diabetes.^19–21^ Further real-time PCR and western blot confirmed the downregulation of cardiac Nur77 in *db/db* mice treated with FGF1^ΔHBS^ (Fig. 5b, c). Nur77 activating Drp1 and translocation to mitochondrial membrane are required for mitochondrial fission.^21^ As shown in Fig. 5d, FGF1^ΔHBS^ treatment reduced the levels of Drp1 and Nur77 in both mitochondria and cytoplasm fractions, and subsequently decreased the release of cytochrome C (Cyt C) into the cytoplasm.

It has been reported that AMPK can activate SIRT1, of which overexpression can further downregulate the mRNA level of Nur77 via deacetylation of Creb.^22–24^ Interestingly, our data showed that FGF1^ΔHBS^ treatment significantly increased cardiac ACC and AMPK phosphorylation accompanied with increased expression of SIRT1 (Fig. 5e) accompanied with enhanced fatty acid oxidation in cardiomyocytes (Supplemental Fig. S4). Taken together, AMPK signaling activation and Nur77 downregulation may mediate the role of FGF1^ΔHBS^ in maintaining cardiac mitochondrial homeostasis.

### FGF1^ΔHBS^ preserved mitochondrial function through AMPK-mediated Nur77 suppression

To testify the role of AMPK and Nur77 in FGF1^ΔHBS^-preserved mitochondrial homeostasis and function, mitochondrial morphology was firstly evaluated in primary neonatal rat cardiomyocytes in the presence or absence of AMPK inhibitor or Nur77 agonist. Heathy mitochondria exhibited tubular structure while mitochondria under stress showed fragmentation and round shape (Supplemental Fig. S5a). Our data revealed that HG led to remarkable mitochondria fragmentations (average length: 2.83 μm) compared to osmotic controls (average length: 7.97 μm) in primary neonatal rat cardiomyocytes (Supplemental Fig. S5a). FGF1^ΔHBS^ treatment completely abrogated HG-induced mitochondrial fragmentation, and this protective effect of FGF1^ΔHBS^ was blocked with either AMPK antagonist (Dors) or Nur77 agonist (CsnB) (Supplemental Fig. S5a). The mitochondrial dynamics was in line with the changes in intracellular ROS and mROS levels (Supplemental Fig. S5b, c). Cardiolipin (CL) is pivotal for oxidative phosphorylation whereas its oxidation by ROS is associated with membrane potential disruption and mitochondrial dysfunction.^25,26^ We found that the protective role of FGF1^ΔHBS^ on oxidative damage of CL was suppressed by AMPK inhibition or Nur77 activation (Supplemental Fig. S5d), which was in line with the alteration of mitochondrial electrochemical gradient (Δψ_m_) (Supplemental Fig. S5e, f).

Subsequently, functional assays using primary cardiomyocytes showed that both basal and maximal respiration rates as well as ATP generation were reduced with HG treatment, which were restored with FGF1^ΔHBS^ treatment (Supplemental Fig. S5g–j). AMPK antagonist or Nur77 agonist blocked these beneficial effects of FGF1^ΔHBS^ (Supplemental Fig. S5g–j). As shown in Supplemental Fig. S5k, HG treatment decreased the phosphorylation of AMPK and expression of SIRT1, Nrf2 and SOD2 but enhanced expression of Nur77 and Drp1, which were restored to normal levels with FGF1^ΔHBS^ treatment. Notably, AMPK antagonist abolished the effect of FGF1^ΔHBS^ on restoring Nur77 expression (Supplemental Fig. S5k). Furthermore, activation of Nur77 using CsnB significantly dampened the beneficial effects of FGF1^ΔHBS^ on AMPK phosphorylation and mitochondrial function in primary cardiomyocytes (Supplemental Fig. S5k).

**Fig. 6:**
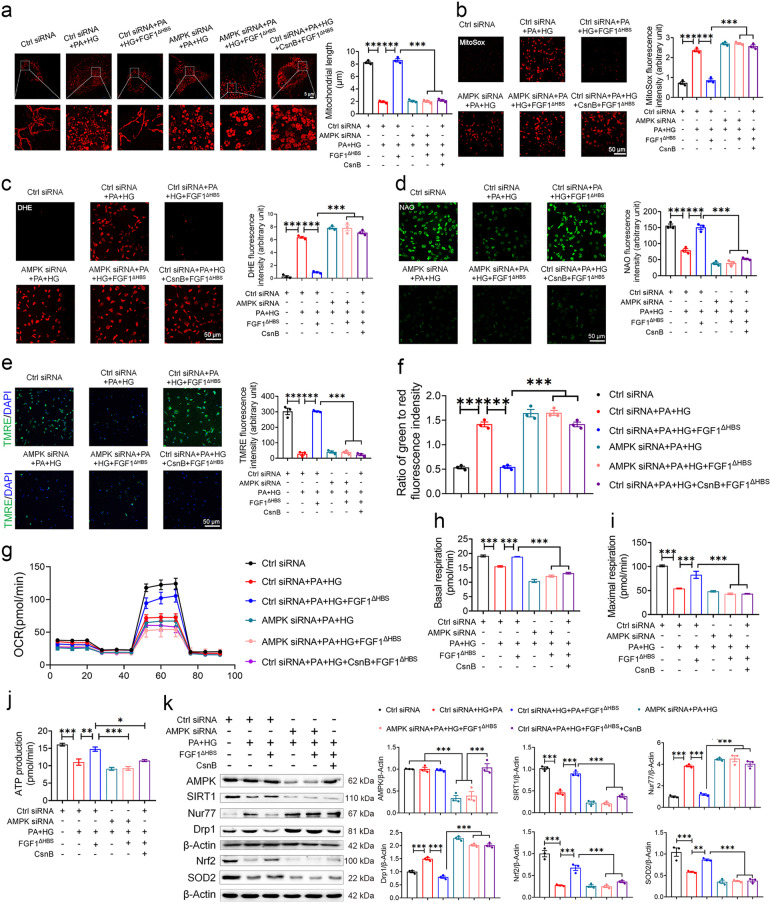
FGF1^∆HBS^ attenuates mitochondrial dysfunction in primary cardiomyocytes via AMPK-Nur77 pathway. **a**–**k** Primary cardiomyocytes were transfected with control or AMPK siRNA and starved for 12 h, and then cardiomyocytes were treated with palmitate (500 μM) and high glucose (35 mM) with or without CsnB (10 μg/mL) in FBS free medium for 1 h, followed by incubation with FGF1^∆HBS^ (500 ng/mL) for additional 48 h. Mannitol + control siRNA group was an osmotic control. **a** Representative images of MitoTracker staining (left panel) and mitochondrial length (right panel) of primary cardiomyocytes. **b**–**d** Representative images of MitoSox (**b**), DHE (**c**), and 10-N-nonyl acridine orange (NAO) (**d**) staining and corresponding quantitative analysis of fluorescence intensity. **e** Representative images of TMRE staining (left panel) and quantitative analysis (right panel) of fluorescence intensity. **f** Mitochondrial membrane potential was evaluated by the ratio of JC-10 fluorescence intensities at 529 nm (green) and 590 nm (red). **g**–**j** Mitochondrial respiratory function was assessed by OCR assay. **k** Western blot analysis (left panel) and densitometric quantification (right panel) of AMPKα2, SIRT1, Nur77, Drp1, Nrf2 and SOD2 in the cardiomyocytes. *n* = 3 independent experiments for each group. Data were mean ± SEM; *P < 0.05, **P < 0.01, ***P < 0.001

To mimic hyperglycemic and hyperlipidemia conditions in vivo, primary neonatal rat cardiomyocytes were treated with palmitate in combination with high glucose (PA and HG). As shown in Fig. 6a–c, AMPKα2 knockdown abolished the inhibitory effects of FGF1^∆HBS^ on PA + HG-induced mitochondrial fission, mROS and intracellular ROS production. In addition, PA and HG also led to other abnormities including decreased mitochondrial respiratory function and increased Nur77 and Drp1 expression, and the protective effects of FGF1^ΔHBS^ were abolished by AMPKα2 knockdown (Fig. 6d–k). Taken together, these results suggest that FGF1^ΔHBS^ preserved mitochondrial function through AMPK-mediated Nur77 suppression in HG with/without PA-treated cardiomyocytes.

### AMPK mediated the protective effect of FGF1^ΔHBS^ against DCM in vivo

**Fig. 7:**
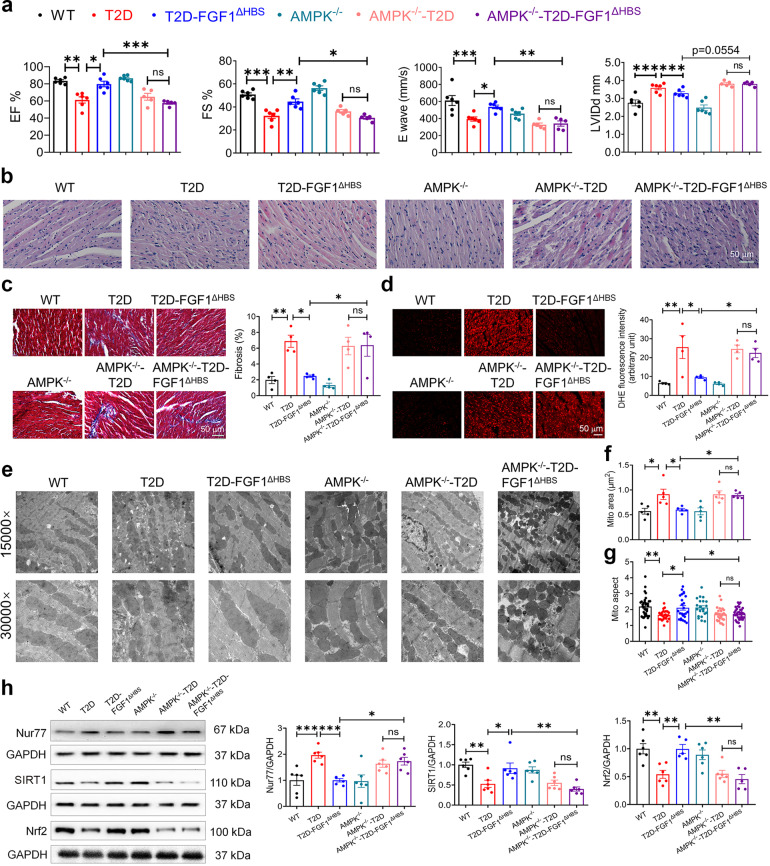
AMPK mediates the protective effect of FGF1^ΔHBS^ against DCM in vivo. **a**–**h** At the age of 8 weeks, male wild type (WT) or AMPKα2 knockout mice were fed with a high-fat diet for 8 weeks and intraperitoneal injected with STZ, and then treated with FGF1^∆HBS^ (0.5 mg/kg body weight) or saline every other day for 8 weeks. **a** Echocardiographic assessment for each group. *n* = 5–6. **b** H&E staining of the cardiac tissues. **c** Masson’s trichrome staining (left panel) and quantitative analysis (right panel) of cardiac fibrosis area. *n* = 4. **d** Representative images (left panel) and quantification (right panel) of fluorescence intensity of DHE in cardiac tissues. *n* = 4. **e** Representative transmission electron micrographs of the cardiac tissues. **f** Mitochondrial area of the cardiac tissues. *n* = 5. **g** Mitochondrial aspect ratio (long/short axis) of the cardiac tissues. *n* = 21–37. **h** Western blot analysis (left panel) and densitometric quantification (right panel) of Nur77, SIRT1 and Nrf2 in the cardiac tissues. *n* = 5–6. Data were mean ± SEM; *P < 0.05, **P < 0.01, ***P < 0.001; ns, not significant

To further confirm whether AMPK activation mediated the protective effects of FGF1^ΔHBS^ against DCM, wild type (WT) and AMPKα2 (the main cardiac catalytic isoform^27,28^) knockout mice (AMPKα2^−/−^) (Supplemental Fig. S6a) were subjected to the DCM model established by HFD feeding plus STZ injection. The random blood glucose level of FGF1^∆HBS^ treated group was ~30% lower than the vehicle-treated group, and lower blood glucose levels were observed in FGF1^∆HBS^ treated group throughout the GTT test. However, the glucose-lowering and insulin-sensitizing effects of FGF1^∆HBS^ were absent in AMPK KO mice (Supplemental Fig. S6b, c). As expected, systolic and diastolic dysfunctions, myofilament disarray, and collagen deposition were comparably observed in WT- and AMPKα2^−/−^-T2D mice (Fig. 7a–c, Supplemental Fig. S6d, and Supplemental Table S2). FGF1^ΔHBS^ treatment improved these cardiac structural alterations, fibrosis and cardiac dysfunction in the WT-T2D mice but not in the AMPKα2^−/−^-T2D mice (Fig. 7a–c).

Similarly, FGF1^ΔHBS^ treatment decreased cardiac ROS level in the WT-T2D mice but not in the AMPKα2^−/−^-T2D mice (Fig. 7d). Further ultrastructural analysis showed that mitochondrial dynamics and morphology in cardiac tissues were improved with FGF1^ΔHBS^ treatment in WT-T2D mice but not in the AMPKα2^−/−^-T2D mice (Fig. 7e–g). Finally, decreased SIRT1 and Nrf2 and increased Nur77 levels were observed in the heart of T2D mice, which were normalized with FGF1^ΔHBS^ treatment in the WT-T2D mice, while these effects of FGF1^ΔHBS^ were abrogated in the AMPKα2^−/−^-T2D mice (Fig. 7h). Taken together, the data indicate that AMPK mediated the protective effects of FGF1^ΔHBS^ on diabetes-induced cardiac dysfunction and mitochondrial injury.

## Discussion

Ample evidence has demonstrated a beneficial role of FGF1 in metabolic diseases, including obesity, type 2 DM, and associated complications.^8,9,29,30^ In this study, we revealed that serum FGF1 was decreased in both T2D patients and mouse models, and FGF1 levels were positively correlated with FS% and negatively correlated with serum BNP in DCM patients. These findings suggested the potential positive correlation between FGF1 levels and cardiac function. However, the potential mitogenic activity of native FGF1 limits in vivo application.^7^ Thus, we engineered an FGF1 variant that lacked the mitogenic property and retained the metabolic effects. The results showed that FGF1^ΔHBS^ treatment significantly prevented cardiac remodeling and restored systolic and diastolic functions in T2D mice (Fig. 2). Our in vitro data revealed that FGF1^ΔHBS^ rescued HG or HG + PA-induced morphological and functional abnormalities in cardiomyocytes (Figs. 1 and 6; and Supplementary Fig. S5), indicating FGF1^ΔHBS^ directly imposes favorable effects on myocardium independent of its glucose-lowering or insulin-sensitizing effects. Further mechanism studies revealed that AMPK-mediated Nur77 suppression and subsequent mitochondrial function improvement played a key role in the therapeutic effects of FGF1^ΔHBS^ on DCM (Figs. 6 and 7).

Mitochondrial dysfunction, oxidative stress and abnormal mitochondrial ultrastructure have been observed in the hearts of both diabetic patients and diabetic animal models, and cause disease progress.^31^ Physiologically mitochondrial fragmentation will increase ATP generation to meet the energetic demand of the heart.^32^ However, excessive mitochondrial fission accentuates Δ*Ψ*m decline, leading to cytochrome c leakage and mitochondrial dysfunction,^33^ which is believed to be the primary source of ROS production and the culprit of DCM.^34,35^ Our data confirmed the abnormally mitochondrial morphology changes with excessive fragmentation, increased ROS generation, suppressed ATP generation and respiratory rates in the in vivo and in vitro models. Importantly, FGF1^ΔHBS^ treatment greatly diminished these abnormalities, which may leverage the rescued expression of Nrf2 and its downstream targets in the hearts since Nrf2 is an important transcription factor in detoxification signaling of oxidative stress in DCM.^36^

Further RNA-Seq revealed that FGF1^ΔHBS^ treatment significantly decreased Nur77 mRNA (Fig. 5a). Although the endogenous ligand for Nur77 receptor has not been identified, studies have revealed Nur77 as a malefactor aggravating cardiac damage by promoting mitochondrial fragmentation, altering mitochondrial membrane potential, and inducing apoptosis through cytochrome c release.^21,37,38^ In the current study, increased Nur77 expression along with impaired mitochondrial oxidative respiration were observed in the cardiac tissues of diabetic mice. Interestingly, FGF1^ΔHBS^ treatment significantly reduced Nur77 and Drp1 expression in vivo and in vitro, which were blunted with AMPK inhibition, indicating an AMPK-mediated Nur77 suppression after FGF1^ΔHBS^ treatment. Furthermore, Nur77 agonist and AMPK siRNA or knockout abolished the beneficial effects of FGF1^ΔHBS^ on HG with/without PA-induced cardiomyocyte dysfunction (Fig. 6) or DCM in vivo (Fig. 7), which indicates an AMPK activation/Nur77 suppression/mitochondrial homeostasis pathway under FGF1^ΔHBS^ treatment.

However, it has been reported that direct pharmacological activation of AMPK in neoplastic cells promotes mitochondrial fission via increasing phosphorylation of MFF, a membrane receptor for Drp1.^39^ This discrepancy may be because of the diverse of metabolic reprogramming between cancer and diabetes and distinct mechanisms for AMPK activation by FGF1^ΔHBS^ and chemicals. Studies by Hall and colleagues have suggested that the AMPK agonist AICAR suppresses IFNγ/TNFα-induced muscle wasting, an effect absent for metformin.^40^ In addition, metformin activates AMPK through inhibition of complex I of the electron transport chain,^41^ the expression of complex I, however, was increased with FGF1^ΔHBS^ treatment. Of note, more studies are necessary to explore the underlying mechanisms of AMPK activation by FGF1^ΔHBS^.

Although hyperglycemia and insulin resistance exacerbate DCM progress, the relevance between blood glucose control and the alleviation of cardiovascular disease is limited.^42^ Previous studies have concluded that hypoglycemic effect alone is not able to retard the onset and progress of diabetic cardiac complications.^43^ The association between insulin-treated DM and worse outcomes of heart failure has been observed.^44^ In addition, anti-glycemic agents such as GLP-1R agonist and DPP-4 inhibitors did not show beneficial effect on reducing the risk of heart failure in clinical trials.^45^ In addition to the anti- hyperglycemia effects, our in vitro data demonstrated directly protective effects of FGF1^ΔHBS^ on cardiomyocyte hypertrophy, apoptosis, mitochondrial homeostasis and oxidative stress. The preservation of mitochondrial dynamics and function could directly improve cardiac function in vivo.^34,46,47^ Therefore, the protective effects of FGF1^ΔHBS^ on DCM are likely mediated by its direct action on mitochondrial homeostasis.

In summary, cardiac injury in diabetic individuals was associated with mitochondrial dysfunction and ROS accumulation caused by upregulated Nur77 and mitochondrial translocation of Drp1 and subsequently excessively mitochondrial fragmentation. Activation of AMPK by the non-mitogenic FGF1^ΔHBS^ greatly reduced Nur77 level, restored mitochondrial function, which protect against myocardial remodeling and dysfunction in diabetes. Given the favorable metabolic activity and reduced proliferative potential, our data offer promise that FGF1^ΔHBS^ may yield a clinically useful agent for the treatment of DCM.

## Materials and methods

### Protein expression and purification

Expression and purification of FGF1^∆HBS^ were performed as previously described.^15^ Briefly, three amino acids of heparin binding sites of FGF1 were mutated as Lys127Asp, Lys128Gln and Lys133Val. Expression of FGF1^∆HBS^ was in competent BL21 (DE3) *Escherichia coli* cells. Following incubation with isopropyl-L-thio-B-D-galactopyranoside (IPTG, 1.0 mM), cells were collected, homogenized, and purified using cation exchange column and size exclusion chromatography. The purity of recombinant FGF1^∆HBS^ protein was estimated to be >98%.

### Human subjects

Human subjects were screened with electrocardiogram and cardiac enzymes analysis. T2D and DCM patients were excluded if they had coronary artery disease, hypertrophic cardiomyopathy, restrictive cardiomyopathy, dilated cardiomyopathy, arrhythmogenic right ventricular dysplasia and other micro- or macrovascular complications of diabetes. In addition, subjects had other comorbidities such as renal failure, severe psychiatric disorders and malignancy were excluded as well. Finally, 17 healthy subjects, 12 T2D patients and 10 T2D patients with DCM were enrolled in the study. Blood samples were obtained from each subject and prepared for FGF1 concentration measurement. The study was conducted in accordance with the principles of the Declaration of Helsinki and approved by the Research Ethics Committee of Wenzhou Medical University. Informed consents were obtained from all recruited subjects. The clinical information was provided in Supplemental Table S3.

### Animals

Eight-week-old male *db/db* (C57BLKS/J-*lepr*^*db*^*/lepr*^*db*^) mice, the non-diabetic *db/m* littermates, and male C57BL/6J mice were purchased from the Model Animal Research Center of Nanjing University (Nanjing, China). Animals were acclimatized for 2 weeks prior to experimentation. The *db/db* mice were employed as a T2D model and were intraperitoneally (IP) injected with FGF1^∆HBS^ (0.5 mg/kg body weight) every other day for 16 weeks from 10 weeks of age. The *db/m* and *db/db* control groups received 0.9% saline accordingly.

AMPKα2 knockout mice were obtained from Dr. Louise D. McCullough at University of Texas Health Science Center at Houston (UTHealth) (Houston, TX) that were originally generated and characterized by Dr. Benoit Viollet at Université Paris Descartes, CNRS (UMR 8104), Paris, France.^48^ C57BL/6J mice and AMPKα2 knockout mice at 8-week-old were fed a high-fat diet (60 % fat; Research Diets, Inc, New Brunswick, NJ) for 8 weeks followed with intraperitoneal injection of streptozotocin (STZ, 35 mg/kg body weight) for 3 consecutive days. And then mice were treated with FGF1^∆HBS^ (IP, 0.5 mg/kg/every other day) or saline for another 8 weeks.

Blood glucose levels were measured using the Precision G Blood Glucose Testing System (Abbott Laboratories, Abbott Park, IL). Plasma insulin levels were determined using a commercial kit (cat #EZRMI-13K, Millipore) according to the manufacture’s instruction. Following the final dose, IPGTT were conducted after fasting overnight (12 h), mice were challenged with a glucose solution (2 g/kg body weight, IP), and their blood were collected at 0, 15, 30, 60, and 120 min post-injection, and the blood glucose levels were determined as above. Area under the curve (AUC) for IPGTT was calculated by applying the trapezoid rule for the glucose tolerance curve using GraphPad Prism.

At the end of the treatment period, mice were evaluated for cardiac function. Mice were then sacrificed, and heart tissues were harvested for further study. All experiments were approved by Wenzhou Medical University Institutional Animal Care and Use Committee.

### Mitochondria isolation

The mitochondrial fraction was prepared using a commercial kit (cat# SM0020, Solarbio, China) in accordance with the manufacturer’s protocol.

### Endogenous FGF1 assay

#### Human sera

Blood samples were left at room temperature for 30 min for clotting prior to centrifugation at 1000 × *g* for 20 min, and serum samples were collected. A human FGF1 ELISA kit (cat# E0032h, EIAab, China) was used to determine the serum FGF1 content in accordance with the manufacturer’s protocol.

#### Mouse sera

Blood samples were left at room temperature for 30 min for clotting prior to centrifugation at 3000 × *g* for 10 min, and serum samples were collected. A mouse FGF1 ELISA kit (cat# P0032Rb-m, EIAab, China) was used to determine the serum FGF1 content in accordance with the manufacturer’s protocol.

### Echocardiography

Systolic and diastolic function were determined using non-invasive transthoracic echocardiography in anesthetized mice one day prior to sacrifice (VEVO3100, Fujifilm Visualsonics).^49^ Left ventricular (LV) dimensions, end-diastolic LV posterior wall thickness (LVPW: d), LV fractional shortening (FS), E waves and LV ejection fraction (EF) were measured. Tei index was calculated based on Doppler recordings of isovolumetric relaxation time (IRT), isovolumetric contraction time (ICT) and ejection time (ET) as follows: TEI = (IRT + ICT)/ET.

### Gene expression analysis

Differential gene expression was evaluated by RNA sequencing (transcriptome sequencing; RNA-Seq). Heart tissues from *db/db* and *db/db* + FGF1^∆HBS^ groups (3 mice per condition) were snap frozen in liquid nitrogen for further analyses. The mRNA was isolated and enriched by magnetic beads with oligo (dT). Sequencing libraries were constructed and sequenced on an Illumina HiSeq 2000 (50 nt single end). Insert sizes in complementary DNA (cDNA) libraries were analyzed using an Agilent 2100 Bioanalyzer. Trimmomatic was chosen to further analyze the raw RNA-Seq. Raw sequencing data were submitted to GEO (GSE153444).

For real-time PCR analysis, total RNA was extracted using an EasyPure RNA kit (cat# ER101-01, Transgen, Beijing, China) and was reverse transcribed with a HiScript II 1st Strand cDNA Synthesis kit (cat# R212-01, Vazyme, Nanjing, China). Real-time PCR was performed using a QuantStudio3 system with AceQ Universal SYBR qPCR Master Mix (cat# Q511-02, Vazyme, Nanjing, China). Primer sequences were listed in Supplemental Table S4.

### Measurement of ROS, NADP^+^, CK-MB

Frozen tissue sections (5 µm in thickness) or cells were incubated with fresh PBS containing dihydroethidium (DHE) or 2’,7’- Dichlorofluorescin diacetate (DCFH-DA) (Beyotime, Nanjing, China) for detection of ROS. A cohort of cells was directly viewed under a fluorescence microscope (Leica, German). Levels of NADP^+^, an index of free radical production, were determined using a commercial kit (cat #G9081, Promega, US) according to the manufacturer’s instruction. CK isoenzyme-MB (CK-MB) in mouse serum was determined using an automatic biochemical analyzer.

### Cell shortening and re-lengthening

Cell shortening and re-lengthening were assessed using a SoftEdge MyoCam® system (IonOptix Corporation, Milton, MA, USA).^50^ Briefly, hearts from adult C57 BL/6J mice were removed and perfused after ketamine/xylazine (ketamine 80 mg/kg and xylazine 12 mg/kg, i.p.) sedation. After digestion with Liberase Blendzyme 4 (Hoffmann-La Roche Inc., Indianapolis, IN), left ventricles were removed, minced and filtered. And then cardiomyocytes were placed in a chamber mounted on the stage of an inverted microscope (IX-70, Olympus). The following indices were analyzed to assess cell mechanics: peak shortening (PS), maximal velocities of cell shortening and re-lengthening (+d*L*/d*t*, −d*L*/d*t*), time-to-PS (TPS) and time-to-90% re-lengthening (TR_90_).

### Apoptosis analysis

TUNEL staining was used to assess apoptosis level according to the manufacturer instruction (cat# 11684795910, Roche, Mannheim, Germany) and counter stained with DAPI. Images were acquired by a confocal microscope (Leica, DE).

### Neonatal cardiomyocytes culture and treatment

Primary cardiomyocytes were prepared from neonatal Sprague Dawley rat hearts and were cultured as previously described.^51^ Briefly, rats (0.5–2 days) were sacrificed by anesthesia (isoflurane). The hearts were harvested and treated with 0.08% trypsin for dissociation of heart tissue. The cells were plated for 1 h into 100-mm culture dishes in DMEM containing 1 g/L of D-glucose supplemented with 10% FBS, 100 U/mL penicillin and 100 mg/mL streptomycin. Suspended cells were then collected as cardiomyocytes, plated in six-well plates or 35-mm culture dishes, and maintained at 37 °C in a humidified 5% CO_2_ incubator for subsequent studies. Cells were starved for 12 h and randomized into different experimental groups: 35 mM d-glucose (Sigma, St. Louis, MO, USA) with or without 500 μM palmitate (Sigma, St. Louis, MO, USA) as PA + HG or HG group, identical concentrations of mannitol as an osmotic control group containing 5.5 mM d-glucose plus 29.5 mM mannitol in the presence or absence of FGF1^WT^/FGF1^∆HBS^ (500 ng/mL) for additional 48 h. For AMPK inhibitory assays, dorsomorphin 2HCl (Dors, Compound C, cat# S7306, Selleck Chemicals) was added 1 h prior to FGF1^∆HBS^ treatment at 10 μM. To activate Nur77, cytosporone B (CsnB, cat# S6674, Selleck Chemicals) was added 1 h prior to FGF1^∆HBS^ treatment at 10 μg/mL.

### Small-interfering RNA (siRNA) transfection

AMPKα2 siRNA (cat# sc-155985) and negative control siRNA (cat# 12935300, Thermo Fisher) were transfected into primary cardiomyocytes with TransMessenger^®^ transfection reagent (cat# 301525, QIAGEN).

### Western blot analysis

Tissues (30–50 mg) or cells were lysed with a RIPA buffer containing protease and phosphatase inhibitors (Thermo Fisher Scientific, MA). Total protein concentrations were determined with the Bradford protein assay kit (Bio-Rad, Hercules, CA). Protein sample aliquots (70 μg) were subjected to electrophoresis, transferred to nitrocellulose membranes, and blocked in Tris-buffered saline containing 0.05% Tween 20 and 5% non-fat dry milk. Primary antibodies used included: FGF1 (1:600, cat# 109180, Abcam), COL 1 (1:1000, cat# ab64883, Abcam), COL 3 (1:1000, cat# ab7778, Abcam), TGF-β1 (1:1000, cat# ab92486, Abcam), cleaved caspase 3 (1:1000, cat# ab2302, Abcam), MyHC (1:1000, cat# ab11083, Abcam), Nrf2 (1:1000, cat# ab62352, Abcam), SOD2 (1:1000, cat# 24127-1-AP, Proteintech), HO-1 (1:1000, cat# 10701-1-AP, Proteintech), Nur77 (1:1000, cat# ab109180, Abcam), OXPHOS (1:600, ab110413, Abcam), Drp1 (1:1000, cat# ab56788, Abcam), Cyt C (1:1000, cat# 10993-1-AP, Proteintech), HSP60 (1:1000, cat# 15282-1-AP, Proteintech), GAPDH (1:1000, cat# 10494-1-AP, Proteintech), β-Actin (1:1000, cat# 60008-1-Ig, Proteintech), CPT-1α (1:1000, cat# 15184-1-AP, Proteintech), PPARα (1:1000, cat# 15540-1-AP, Proteintech), phospho-ACC (1:1000, cat# ab68191, Abcam), ACC (1:1000, cat# ab45174, Abcam), phospho-AMPK (1:1000, cat# 50081, Cell Signaling Technology), AMPK (1:1000, cat# ab3760, Abcam) and SIRT1 (1:1000, cat# ab110304, Abcam). The immunoreactive bands were detected with secondary antibodies (Santa Cruz Biotechnology, Dallas, TX), and bands visualized using the ECL detection kit (Bio-Rad, Hercules, CA). Densitometric analysis was performed using Image J software version 1.38e (NIH, Bethesda, MD).

### Histology and immunofluorescence analysis

Mouse hearts were fixed in 4% paraformaldehyde solution, embedded in paraffin, and sectioned at 5 µm thickness. After dehydration, sections were stained with hematoxylin and eosin (H&E), Masson trichrome staining (Beyotime Biotech, Nantong, China) and Sirius red staining kits (Beyotime Biotech, Nantong, China) per manufacturer’s instruction. Stained sections were evaluated for histopathological damage under light microscopy (×400 magnification; Nikon, Japan). Percentage of fibrosis was analyzed using an Image J software.

For immunofluorescence staining, heart frozen sections (10 μm) or cell samples were fixed with 4% paraformaldehyde for 15 min. Specimens were subsequently incubated with fluorescent probes, including wheat germ agglutinin (WGA, cat# W6748, Thermo Fisher) to indicate cardiomyocyte membrane, TRITC Phalloidin (cat# 40734ES75, Yeasen, Shanghai, China) to visualize F-actin, MitoTracker (cat# M7512, Thermo Fisher) to visualize mitochondria, and 10-N-nonyl acridine orange (cat# A1372, Thermo Fisher) to visualize cardiolipin at room temperature. After washing, samples were observed under a confocal microscope (Leica, Mannheim, Germany or A1R-SIM-STORM, Nikon, Japan).

For electron microscopy analysis, heart samples were fixed using a triple aldehyde fixative for 1 h after harvested from mice, and post-fixed in 1% osmium tetroxide for 2 h. After rinsing with distilled water, specimens were incubated with 0.25% uranyl acetate overnight, dehydrated and embedded in epoxy resin (Epok). Ultrathin sections (~70 nm) were cut, stained with 2% uranyl acetate and Reynolds lead citrate, and examined under an electron microscope (JEOL, JEM-1230, Japan).

### ATP measurement, mitochondrial membrane potential (ΔΨm), and mROS analyses

A commercial assay kit (cat# ab83355, Abcam) was used to determine ATP content per the manufacturer instruction. Mitochondrial membrane potential was determined with a JC-10 kit (cat# J8050, Solarbio, China) and TMRE (cat# T669, Invitrogen). Mitochondrial ROS were evaluated with MitoSox indicator (cat# 9082, Cell Signaling Technology).

### DNA isolation and mtDNA quantification

Total DNA were isolated using a commercial kit (cat# EE101-11, Transgen, Beijing, China) following the manufacture instruction. The mtDNA and nDNA copy numbers were measured as previously described.^52^

### Oxygen consumption rate (OCR) measurement

Mitochondrial oxygen consumption rate (OCR) was evaluated using a XFe96 extracellular flux analyzer (Agilent Technologies) as previously described.^20^ Primary cardiomyocytes were isolated and seeded at 25,000 cells/well on 96-well XFe96 cell culture microplates and cultured for 48 h.

### Statistical analysis

In vitro experiments were repeated in triplicate (biological repeat) for each individual experiment. All data were expressed as mean ± SEM. For the comparisons of two groups, two-tailed unpaired Student’s *t*-test was performed. One-way ANOVA followed by Turkey post hoc test was used to compare mean values of more than two groups. The differences between two categorical variables were determined by Fisher’s exact test. Two-way ANOVA followed by Turkey post hoc test was used to compare the effects of AMPK knockout or knockdown in response to FGF1^∆HBS^ treatment. All statistical tests were analyzed by GraphPad Prism. *P*-value < 0.05 was considered statistically significant.

## Supplementary information

Supplementary Materials

## Data Availability

The datasets that support the findings of this study are available from the corresponding author upon reasonable request.
