# Supplementary material for: FGF1ΔHBS prevents diabetic cardiomyopathy by maintaining mitochondrial homeostasis and reducing oxidative stress via AMPK/Nur77 suppression
Source: Signal Transduct Target Ther. 2021 Mar 24;6:133. doi: 10.1038/s41392-021-00542-2 (PMC7991671; doi:10.1038/s41392-021-00542-2)
Supplement: Supplementary file 1 — Supplementary Materials [file 41392_2021_542_MOESM1_ESM.docx]

Supplementary Materials for

FGF1^ΔHBS^ prevents diabetic cardiomyopathy by maintaining mitochondrial homeostasis and reducing oxidative stress via AMPK/Nur77 suppression

Dezhong Wang^1,2#^, Yuan Yin^3#^, Shuyi Wang^4#^, Tianyang Zhao^2#^, Fanghua Gong^2^, Yushuo Zhao^2^, Beibei Wang^2^, Yuli Huang^2^, Zizhao Cheng^2^, Guanghui Zhu^3^, Zengshou Wang^3^, Yang Wang^2^, Jun Ren^4^, Guang Liang^2^*, Xiaokun Li^2^*, Zhifeng Huang^2^*

^1^School of Life and Environmental Science, Wenzhou University, Wenzhou, Zhejiang 325035, China

^2^School of Pharmaceutical Sciences and Center for Structural Biology, Wenzhou Medical University, Wenzhou, Zhejiang 325035, China

^3^The 2^nd^ Affiliated Hospital, Wenzhou Medical University, Wenzhou, Zhejiang 325035, China

^4^Department of Cardiology and Shanghai Institute of Cardiovascular Diseases, Zhongshan Hospital Fudan University, Shanghai, 200032, China.

^#^These authors contributed equally to this work.

*Correspondence to: hzf@wmu.edu.cn (Z.H.); xiaokunli@wmu.edu.cn (X.L.); cuiliang1234@163.com (G.L.)

**This PDF file includes:**

Figures S1-S6

Tables S1-S4


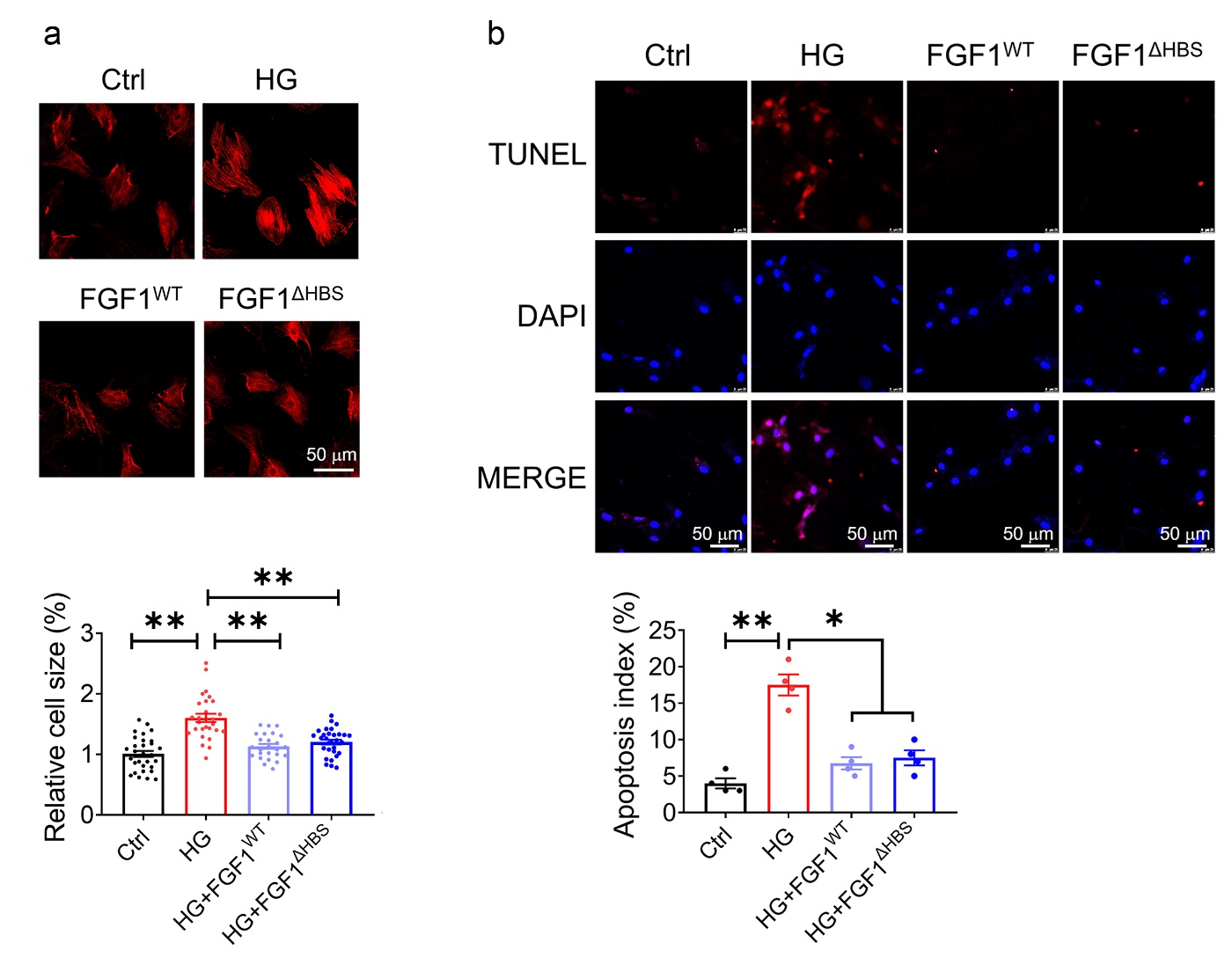


**Fig. S1 FGF1^ΔHBS^ prevents high glucose induced hypertrophy and apoptosis in neonatal rat cardiomyocytes.**

**a-b** Primary cardiomyocytes were serum starved for 12 h and treated with high glucose (35 mM) at the presence or absence of FGF1^WT^ and FGF1^∆HBS^ (500 ng/mL) for 48 h. Mannitol group as an osmotic control (Ctrl). **a** Representative images (upper panel) and quantification (lower panel) of TRITC-Phalloidin immunofluorescence. n=26-31; 3 independent experiments for each group. **b** Representative images (upper panel) and quantification (lower panel) of TUNEL staining. n=4 independent experiments for each group. Data were mean ± SEM; **P*<0.05, ***P*<0.01.


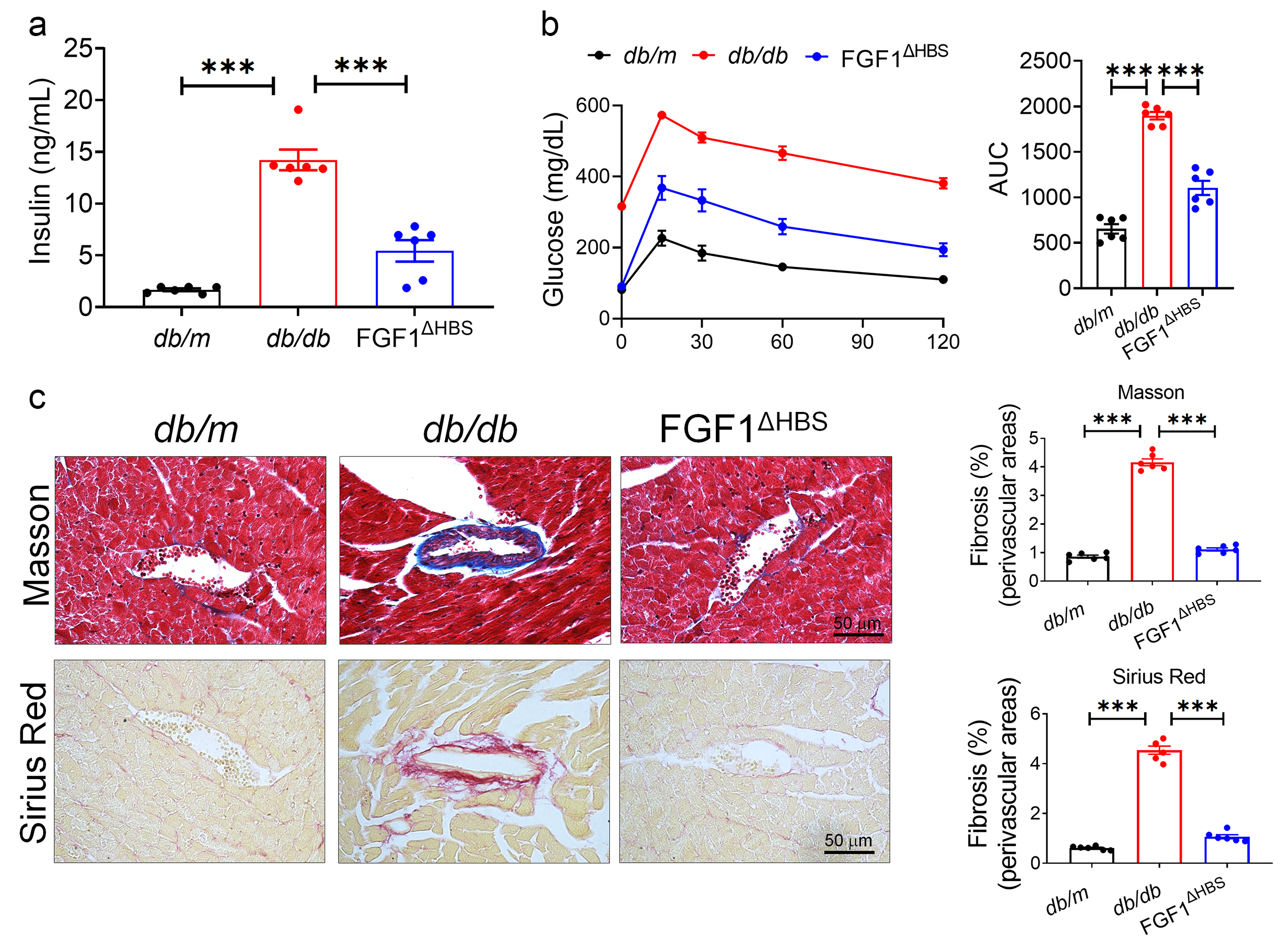


**Fig. S2 FGF1^ΔHBS^ improved insulin sensitization and prevents** **perivascular fibrosis in hearts.**

**a-c** *db/db* mice were treated with FGF1^ΔHBS^ (0.5 mg/kg body weight) or vehicle every other day for 16 weeks. **a** Insulin levels; **b** GTT (left panel) and AUC of GTT (right panel); **c** Masson and Sirius red staining (left panel) and densitometric quantification (right panel) in the cardiac tissues. n=6. Data were mean ± SEM; ****P*<0.001.


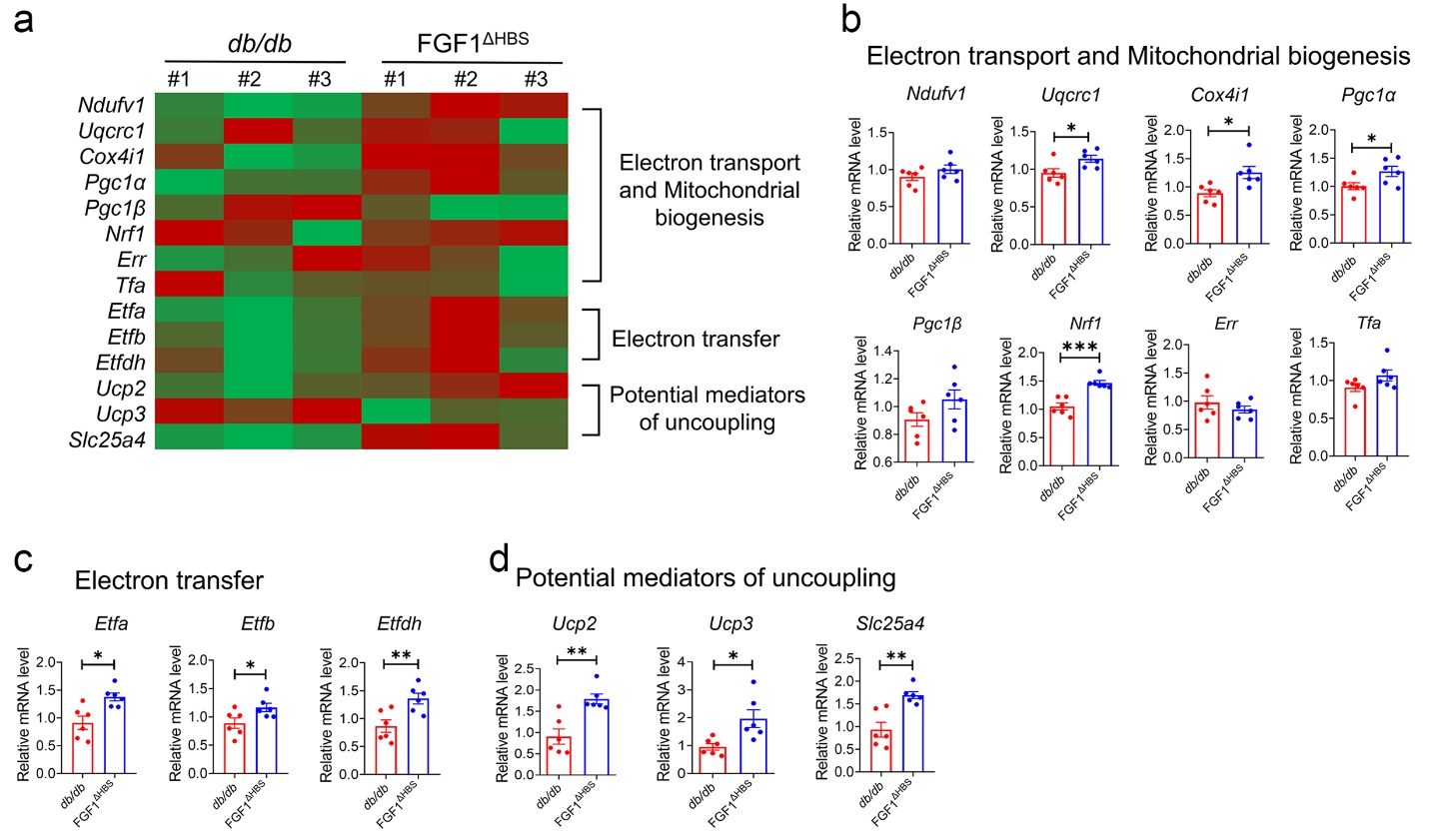


**Fig. S3 FGF1^ΔHBS^ upregulates mitochondrial biogenesis- and OXPHOS-related genes.**

**a-d** *db/db* mice were treated with FGF1^ΔHBS^ (0.5 mg/kg body weight) or vehicle every other day for 16 weeks. **a** Hierarchical clustering of FGF1^ΔHBS^ upregulated genes related to mitochondrial biogenesis and OXPHOS based on the RNA sequencing results. **b-d** The mRNA levels of indicated genes in cardiac tissues of each group. n=6. Data were mean ± SEM; **P*<0.05, ***P*<0.01, ****P*<0.001.


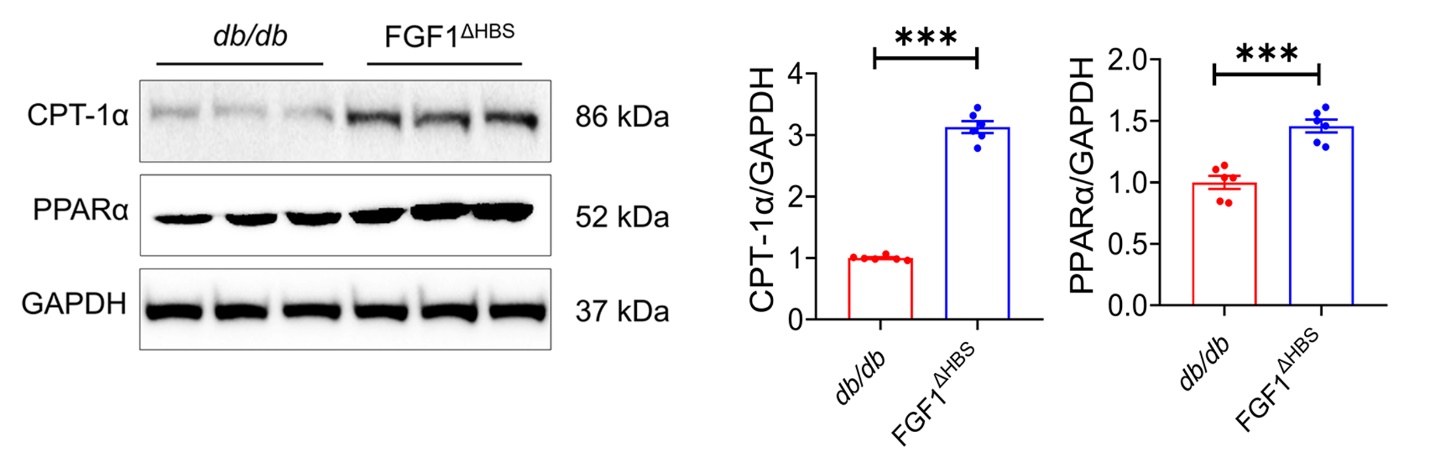


**Fig. S4 FGF1^ΔHBS^ enhances FAO.**

Western blot analysis (left panel) and densitometric quantification (right panel) of CPT-1α and PPARα of *db/db* mice treated with FGF1^ΔHBS^ (0.5 mg/kg body weight) or vehicle every other day for 16 weeks. GAPDH was a loading control. n=6. Data were mean ± SEM; ****P*<0.001.


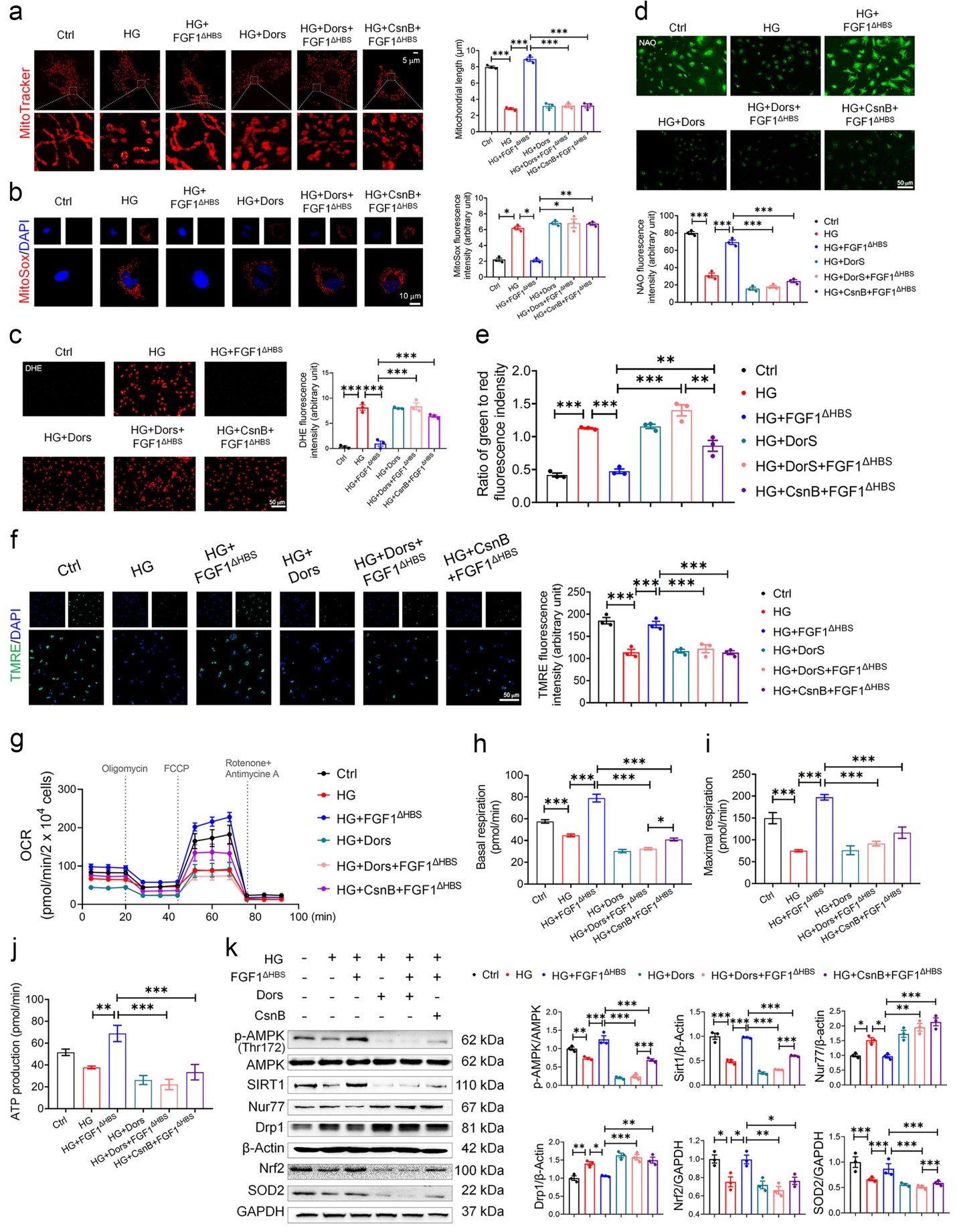


**Fig. S5 FGF1^∆HBS^ attenuates high glucose-induced mitochondrial dysfunction via AMPK/Nur77 signal transduction.**

**a-k** Primary cardiomyocytes were serum starved for 12 h and treated with high glucose (35 mM) with or without Dors (10 μM) and CsnB (10 μg/mL) in FBS free medium for 1 h, followed by incubation with FGF1^∆HBS^ (500 ng/mL) for additional 48 h. Mannitol group was an osmotic control. **a** Representative images of MitoTracker staining (left panel) and mitochondrial length (right panel) of primary cardiomyocytes. **b-d** Representative image of MitoSox (**b**), DHE (**c**) and 10-N-nonyl acridine orange (NAO) (**d**) staining and quantitative analysis of the fluorescence intensities of each group. **e** Representative images of TMRE staining (left panel) and quantitative analysis (right panel) of fluorescence intensity. **f** Mitochondrial membrane potential was evaluated by the ratio of JC-10 fluorescence intensities at 529 nm (green) and 590 nm (red). **g-j** Mitochondrial respiratory function was assessed by OCR assay. **k** Western blot analysis (left panel) and densitometric quantification (right panel) of p-AMPK, AMPKα2, SIRT1, Nur77, Drp1, Nrf2 and SOD2 in the cardiomyocytes. n=3 independent experiments for each group. Data were mean ± SEM; **P*<0.05, ***P*<0.01, ****P*<0.001.

**
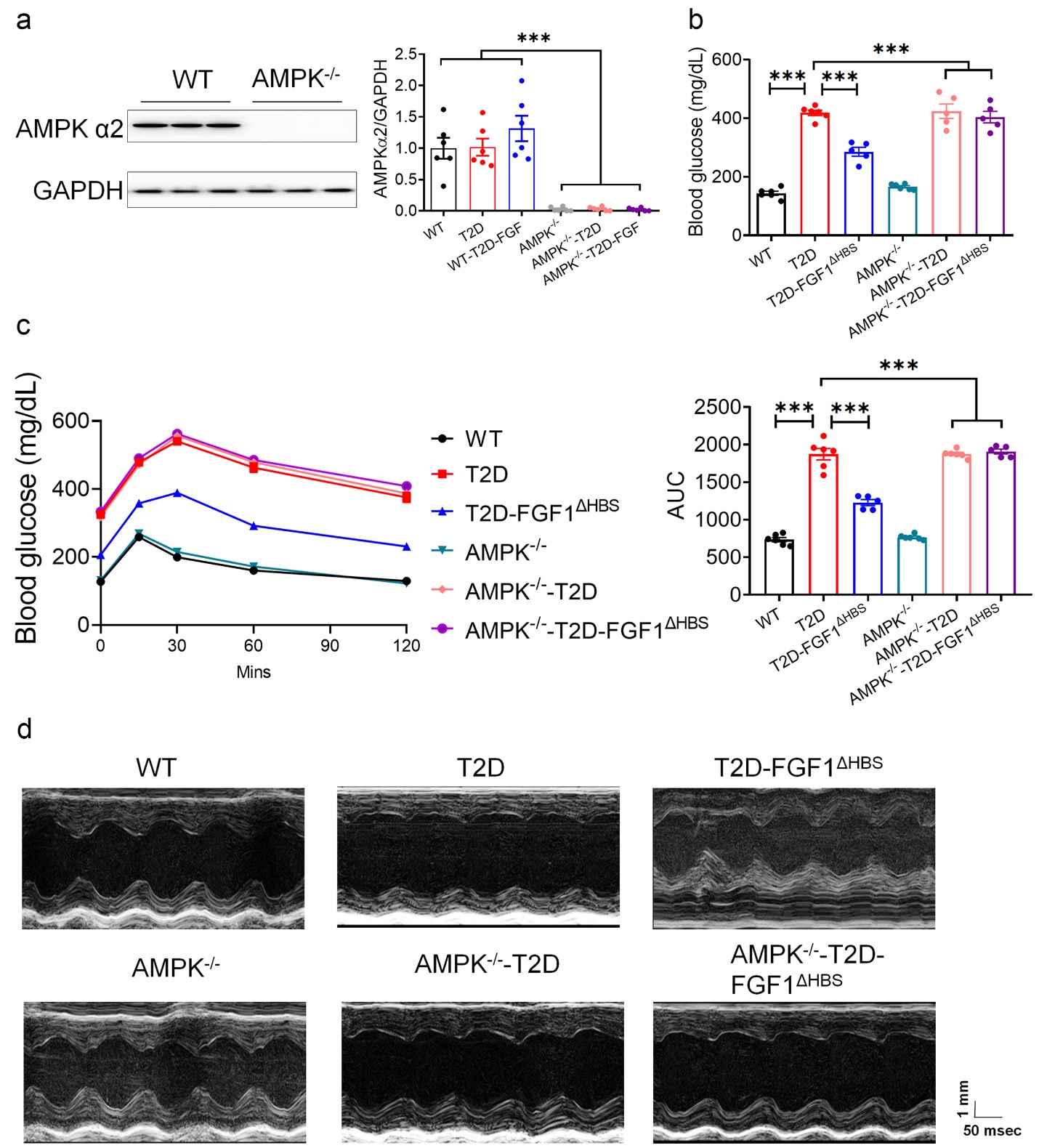
**

**Fig. S6 Glucose lowering and cardioprotection effects of FGF1^∆HBS^ was AMPK dependent.**

**a** Western blot analysis (left panel) and densitometric quantification (right panel) of AMPKα2 in the cardiac tissues. n=6. **b** Random blood glucose levels. n=5-6. **c** GTT (left panel) and AUC of GTT (right panel). n=5-6. **d** Representative images of echocardiography. n=5-6. Data were mean ± SEM; ****P*<0.001.

**Table S1** **Protective effects of FGF1^ΔHBS^ on T2D induced cardiac dysfunction.**

|  | *db/m* | *db/db* | *db/db*+FGF1^ΔHBS^ |
| --- | --- | --- | --- |
| Weight, g | 24.97±0.77 | 61.33±3.4* | 49.41±1.568^#^ |
| Glucose, mg/dl | 129±24.3 | 386.1±54.15* | 115.4±9.040^#^ |
| HW/TL (mg/mm) | 6.58±1.75 | 9.577±1.21* | 7.106±1.3336^#^ |
| IVSd (mm) | 0.615±0.007 | 0.676±0.005* | 0.635±0.002 |
| PWd (mm) | 0.808±0.006 | 0.925±0.016* | 0.812±0.005^#^ |
| CK-MB, IU/L | 413.1±117.5 | 1542.5±103.4* | 703.8±110.8^#^ |
| IRT, ms | 18±4.06 | 28.00±5.39* | 15.13±2.031^#^ |
| Tei Index | 0.85±0.29 | 1.229±0.14* | 0.934±0.2034^#^ |

HW, heart weight; TL, tibia length; IVSd, interventricular septal dimension in diastole; PWd, posterior wall thickness in diastole; CK-MB, creatine kinase isoenzyme; IRT, isovolumetric relaxation time; Tei Index, a myocardial performance index designed by Dr Chuwa Tei. n=6. Data were mean ± SEM; **p*<0.05 vs *db/m*; ^#^*p*<0.05 vs *db/db*.

**Table S2** **Protective effects of FGF1^ΔHBS^ on HFD/STZ induced cardiac dysfunction.**

|  | WT | T2D | T2D-FGF1^ΔHBS^ | AMPK^-/-^ | AMPK^-/-^-T2D | AMPK^-/-^-T2D-FGF1^ΔHBS^ |
| --- | --- | --- | --- | --- | --- | --- |
| Weight, g | 25.42±0.63 | 29.78±0.42* | 26.05±1.01^#^ | 24.56±0.61 | 29.44±0.49^$^ | 29.8±0.76^$^ |
| HW/BW | 4.98±0.14 | 5.53±0.12* | 5.09±0.18^#^ | 5.05±0.11 | 5.41±0.23^$^ | 5.32±0.14^$^ |
| IVSd (mm) | 0.707±0.038 | 0.683±0.034 | 0.733±0.023^#^ | 0.713±0.032 | 0.694±0.028 | 0.701±0.025 |
| PWd (mm) | 0.912±0.049 | 0.996±0.044* | 0.905±0.069^#^ | 0.891±0.079 | 1.013±0.034^$^ | 1.023±0.117^$^ |

HW, heart weight; BW, body weight; IVSd, interventricular septal dimension in diastole; PWd, posterior wall thickness in diastole; n=5-6. Data were mean ± SEM; **p*<0.05 vs WT; #*p*<0.05 vs T2D; $*p*<0.05 vs T2D-FGF1^ΔHBS^.

**Table S3 Clinical characteristics of human subjects**

| / | Age  (yr) | Gender | Blood glucose  (Max) | Medications for blood glucose control | Duration of  diabetes (yr) | LVEF, % | BNP (pg/mL) | IVSd (mm) | PWd (mm) | HbA1c, % |
| --- | --- | --- | --- | --- | --- | --- | --- | --- | --- | --- |
| Healthy Subjects | | | | | | | | |  |  |
| 1 | 58 | Male | 4.4 | NO | 0 | 66.5 | 9 | 9 | 10 | 5.1 |
| 2 | 62 | Male | 5.1 | NO | 0 | 70.4 | 12 | 10 | 10 | 5 |
| 3 | 70 | Male | 5.3 | NO | 0 | 68.1 | 7 | 9 | 9 | 5 |
| 4 | 49 | Male | 4.7 | NO | 0 | 72.6 | 22 | 10 | 9 | 5.5 |
| 5 | 54 | Female | 4.5 | NO | 0 | 69 | 30 | 11 | 10 | 5.8 |
| 6 | 57 | Female | 4.5 | NO | 0 | 74.7 | 11 | 10 | 9 | 4.9 |
| 7 | 66 | Male | 5.6 | NO | 0 | 67.8 | 23 | 9 | 9 | 5.2 |
| 8 | 49 | Male | 5.4 | NO | 0 | 75.6 | 20 | 9 | 10 | 5.5 |
| 9 | 44 | Female | 5.8 | NO | 0 | 68.9 | 16 | 9 | 9 | 5.4 |
| 10 | 37 | Male | 5.4 | NO | 0 | 72.3 | 3 | 10 | 9 | 5.8 |
| 11 | 67 | Female | 4.9 | NO | 0 | 78 | 7 | 10 | 10 | 6 |
| 12 | 36 | Female | 5.9 | NO | 0 | 77.2 | 4 | 11 | 10 | 5.5 |
| 13 | 64 | Female | 5.6 | NO | 0 | 79.6 | 15 | 11 | 10 | 5.3 |
| 14 | 34 | Male | 5 | NO | 0 | 69.7 | 13 | 9 | 9 | 5.3 |
| 15 | 59 | Female | 5.2 | NO | 0 | 73.5 | 27 | 10 | 10 | 5 |
| 16 | 53 | Female | 6.1 | NO | 0 | 71.2 | 56 | 10 | 9 | 4.7 |
| 17 | 68 | Female | 5.7 | NO | 0 | 69.4 | 19 | 10 | 9 | 4.9 |
| Mean±  SEM | 54.53±  0.68 | / | 5.24±  0.03 | / | 0 | 72.03±  0.23 | 17.3±  0.75 | 9.82±  0.04 | 9.47±  0.03 | 5.29±  0.02 |
| T2DP |  |  |  |  |  |  |  |  |  |  |
| 1 | 72 | Female | 15.7 | YES | 30 | 60.7 | 96 | 11 | 11 | 5.6 |
| 2 | 85 | Male | 24.1 | YES | 20 | 69 | 290 | 9 | 9 | 11.9 |
| 3 | 48 | Male | 19.4 | YES | 10 | 63.4 | 76 | 10 | 11 | 9 |
| 4 | 56 | Female | 20.3 | YES | 14 | 63.7 | 92 | 9 | 10 | 7.8 |
| 5 | 66 | Female | 17.2 | YES | 10 | 59.9 | 137 | 10 | 10 | 8.3 |
| 6 | 59 | Female | 18.8 | YES | 10 | 69 | 88 | 10 | 9 | 8 |
| 7 | 70 | Female | 22 | YES | 15 | 67.8 | 134 | 10 | 10 | 8.2 |
| 8 | 62 | Male | NA | NO | 3 | 75.1 | 57 | 9 | 9 | 5.3 |
| 9 | 44 | Male | NA | NO | 2 | 72 | 73 | 10 | 10 | 5.1 |
| 10 | 67 | Female | 15.7 | YES | 12 | 65 | 97 | 9 | 11 | 4.9 |
| 11 | 63 | Female | 12 | YES | 11 | 66.2 | 22 | 10 | 11 | 5 |
| 12 | 47 | Male | 9.5 | YES | 8 | 72.3 | 33 | 10 | 10 | 5.5 |
| Mean±  SEM | 61.58±  0.98 | / | 17.47±  0.44* | / | 12.08±  0.62 | 67±  0.39 | 99.58±  5.76 | 9.75±  0.05 | 10.08±  0.07 | 7.05±  0.18* |
| DCM |  |  |  |  |  |  |  |  |  |  |
| 1 | 65 | Male | 11.8 | YES | 20 | 41 | 744 | 11 | 8 | 9.5 |
| 2 | 58 | Male | 13 | YES | 10 | 55.9 | 566 | 12 | 11 | 5.8 |
| 3 | 60 | Male | 14.4 | YES | 8 | 58.8 | 947 | 13 | 13 | 8 |
| 4 | 52 | Female | 12.9 | YES | 10 | 66.5 | 523 | 10 | 10 | 9.1 |
| 5 | 55 | Female | 26.4 | YES | 10 | 73.4 | 210 | 9 | 9 | 6.5 |
| 6 | 81 | Male | 13.1 | YES | 10 | 62 | 631 | 11 | 11 | 6.8 |
| 7 | 72 | Male | 12.8 | YES | 2 | 55.3 | 518 | 10 | 10 | 6.6 |
| 8 | 82 | Male | 10 | YES | 10 | 61 | 478 | 12 | 11 | 6.7 |
| 9 | 67 | Male | 10 | YES | 10 | 61.7 | 509 | 13 | 12 | 13.2 |
| 10 | 57 | Female | 17.8 | YES | 20 | 60 | 561 | 12 | 11 | 9.7 |
| Mean±  SEM | 64.9±  1.06 | / | 14.22±  0.48* | / | 11±  0.54 | 59.56±  0.84*^, #^ | 568.7±  18.94*^, #^ | 11.3±  0.13*^, #^ | 10.6±  0.14* | 8.13±  0.22* |

Data were mean ± SEM; **P*<0.05 vs Healthy subjects, ^#^*P*<0.05 vs T2DP.

**Table S4 Primers used in this study**

| Gene Name | Primer Name | Sequence (5' -> 3') |
| --- | --- | --- |
| Uqcrc1 | mUqcrc1-F | TGCCAGAGTTTCCAGACCTT |
| Uqcrc1 | mUqcrc1-R | CCAAATGAGACACCAAAGCA |
| Ndufv1 | mNdufv1-F | TGTGAGACCGTGCTAATGGA |
| Ndufv1 | mNdufv1-R | CATCTCCCTTCACAAATCGG |
| Cox4i1 | mCOX4-F | CGCTGAAGGAGAAGGAGAAG |
| Cox4i1 | mCOX4-R | GCAGTGAAGCCAATGAAGAA |
| Pgc1α | mPGC-1α-F | GTAAATCTGCGGGATGATGG |
| Pgc1α | mPGC-1α-R | AGCAGGGTCAAAATCGTCTG |
| Pgc1β | mPGC-1β-F | TGAGGTGTTCGGTGAGATTG |
| Pgc1β | mPGC-1β-R | CCATAGCTCAGGTGGAAGGA |
| Nrf1 | mNrf1-F | CTTCAGAACTGCCAACCACA |
| Nrf1 | mNrf1-R | GCTTCTGCCAGTGATGCTAC |
| Errα | mERRα-F | GGAGGACGGCAGAAGTACAA |
| Errα | mERRα-R | CAGGTTCAACAACCAGCAGA |
| Tfa | mTFAm-F | CAAAAAGACCTCGTTCAGCA |
| Tfa | mTFAm-R | CTTCAGCCATCTGCTCTTCC |
| Sod2 | mSOD2-F | ACAACTCAGGTCGCTCTTCA |
| Sod2 | mSOD2-R | GAACCTTGGACTCCCACAGA |
| Ucp2 | mUCP2-F | TCTCCTGAAAGCCAACCTCA |
| Ucp2 | mUCP2-R | CTACGTTCCAGGATCCCAAG |
| Ucp3 | mUCP3-F | TTTGGAGCTGGCTTCTGTG |
| Ucp3 | mUCP3-R | AAGGCCCTCTTCAGTTGCTC |
| Slc25a4 | mSlc25a4-F | TGATTGTGTCGTGAGAATCCCC |
| Slc25a4 | mSlc25a4-R | AGAACTGCTTATGGCGATCCA |
| Etfa | mETFA-F | GCCTCATTGCTCCGTTTTCAG |
| Etfa | mETFA-R | GCTACTAAGCAGGACACTTCAC |
| Etfb | mETFB-F | CTGTCAAGAGGGTCATCGACT |
| Etfb | mETFB-R | CCAGAGCAGTTCGGATGGTC |
| Etfdh | mEtfdh-F | AAAGGGGCTCCACTTAATACTCC |
| Etfdh | mEtfdh-R | CATGATTGTTCATCGGAAGACCT |
| Gsta3 | mGSTA3-F | AAGAATGGAGCCTATCCGGTG |
| Gsta3 | mGSTA3-R | TTGAGTTCGGCAAGCCTACC |
| Nur77 | NR4A1-F | GTGTACCCGTCCATGAAGGTG |
| Nur77 | NR4A1-R | GGCTGGAAGTTGGGTGTAGA |
| β-actin | β-actin-F | CGTCATTGCACGAAGACACAA |
| β-actin | β-actin-R | CCTGGTCCACCATTTTAAGGC |
| Nrf2 | Nfe2l2-F | TCTTGGAGTAAGTCGAGAAGTGT |
| Nrf2 | Nfe2l2-R | GTTGAAACTGAGCGAAAAAGGC |
| Ho1 | HO-1-F | AAGCCGAGAATGCTGAGTTCA |
| Ho1 | HO-1-R | GCCGTGTAGATATGGTACAAGGA |
